# One for all and all for One: Improving replication of genetic studies through network diffusion

**DOI:** 10.1371/journal.pgen.1007306

**Published:** 2018-04-23

**Authors:** Daniel Lancour, Adam Naj, Richard Mayeux, Jonathan L. Haines, Margaret A. Pericak-Vance, Gerard D. Schellenberg, Mark Crovella, Lindsay A. Farrer, Simon Kasif

**Affiliations:** 1 Bioinformatics Graduate Program, Boston University, Boston, Massachusetts, United States of America; 2 Department of Medicine (Biomedical Genetics), Boston University School of Medicine, Boston, Massachusetts, United States of America; 3 Department of Pathology and Laboratory Medicine, University of Pennsylvania, Philadelphia, Pennsylvania, United States of America; 4 Department of Neurology and Sergievsky Center, Columbia University, New York, New York, United States of America; 5 Department of Epidemiology and Biostatistics, Case Western Reserve University, Cleveland, Ohio, United States of America; 6 Hussman Institute for Human Genomics, University of Miami Miller School of Medicine, Miami, Florida, United States of America; 7 Department of Computer Science, Boston University, Boston, Massachusetts, United States of America; 8 Department of Neurology, Boston University School of Medicine, Boston, Massachusetts, United States of America; 9 Department of Ophthalmology, Boston University School of Medicine, Boston, Massachusetts, United States of America; 10 Department of Biostatistics, Boston University School of Public Health, Boston, Massachusetts, United States of America; 11 Department of Epidemiology, Boston University School of Public Health, Boston, Massachusetts, United States of America; 12 Department of Biomedical Engineering, Boston University, Boston, Massachusetts, United States of America; Stanford University School of Medicine, UNITED STATES

## Abstract

Improving accuracy in genetic studies would greatly accelerate understanding the genetic basis of complex diseases. One approach to achieve such an improvement for risk variants identified by the genome wide association study (GWAS) approach is to incorporate previously known biology when screening variants across the genome. We developed a simple approach for improving the prioritization of candidate disease genes that incorporates a network diffusion of scores from known disease genes using a protein network and a novel integration with GWAS risk scores, and tested this approach on a large Alzheimer disease (AD) GWAS dataset. Using a statistical bootstrap approach, we cross-validated the method and for the first time showed that a network approach improves the expected replication rates in GWAS studies. Several novel AD genes were predicted including *CR2*, *SHARPIN*, *and PTPN2*. Our re-prioritized results are enriched for established known AD-associated biological pathways including inflammation, immune response, and metabolism, whereas standard non-prioritized results were not. Our findings support a strategy of considering network information when investigating genetic risk factors.

## Author summary

Integrating multiple types of -omics data is a rapidly growing research area due in part to the increasing amount of diverse and publicly accessible data. In this study, we demonstrated that integration of genetic association and protein interaction data using a network diffusion approach measurably improves reproducibility of top candidate genes. Application of this approach to Alzheimer disease (AD) using a large dataset assembled by the Alzheimer’s Disease Genetics Consortium identified several novel candidate AD genes that are supported by pre-existing knowledge of AD pathobiology. Our findings support a strategy of considering network information when investigating genetic risk factors. Finally, we developed a transparent and easy-to-use R package that can facilitate the extension of our methodology to other phenotypes for which genetic data are available.

## Introduction

The discovery of disease-associated genomic variation has numerous clinical and scientific applications, including earlier disease prognosis, improved understanding of disease pathophysiology, and development of personalized treatment therapies [1]. A commonly used technique for identifying these mutations is the genome wide association study (GWAS) approach [2]. Typically, a large sample of affected and unaffected individuals are genotyped for many single nucleotide polymorphisms (SNPs) using a high-density microarray chip and then test statistically if the allele frequency of each variant is associated with disease status [2]. Significant associations in this first step (“discovery phase”) are deemed to be robust if they replicate in an independent cohort (“replication phase”). In this study, we focused on improving the replicability of GWAS results for Alzheimer disease (AD), although our methodology is applicable to genetic data for other diseases and traits. AD is a neurodegenerative disease resulting in irreversible dementia and memory loss with elevated prevalence in older populations [3]. Recent estimates suggest that approximately 5.4 million Americans have AD, and the number of cases of AD is expected to increase dramatically in future years if medical advances continue to improve life expectancy, thereby allowing more individuals to reach ages where AD is on the rise [3].

Genetic studies of AD have led to identifying numerous AD associated genes such as *APP* [4], *PSEN1* [5], and *PSEN2* [6] for early onset AD (EOAD), as well as *APOE* [7, 8] and *SORL1* [8, 9] for late onset AD (LOAD). Common variants in more than 20 other genes have been robustly associated with AD risk [8]. However, not all AD associated genes will reach genome wide significance in current datasets of sample sizes below 100,000 individuals. It is well recognized that incorporating other forms of biological data improves confidence in genetic findings [10–12].

Our computational framework is based on the following biological hypothesis. If a known AD variant is associated with a gene that is involved in a particular biological process (BP) (e.g. inflammation), we assume as a probabilistic prior that other AD variants might be associated with proteins involved in this BP or proteins that physically interact with this BP. This hypothesis can be tested computationally using a protein interaction network [13–15] by extending the “guilt by association” principle via propagation of probabilistic evidence in a network [16, 17]. This general idea has similarity to the Google ranking algorithm of web pages, in which a web page that has a short link distance to many “important” pages will itself be considered “important”.

In the case of protein interactions, guilt by association-based inference is typically performed by inspecting the function of direct neighbors of a predicted disease gene in a protein-interaction network. This approach has been incorporated in multiple interpretation systems as well as commercially such as Ingenuity Pathway Analysis (IPA). However, it has been shown that network propagation, diffusion or other related methods that go beyond simple neighbor-based analysis can carry functional or disease associations further in the network with improved predictive accuracies [10, 11]. This idea extends to predicting both gene function and disease phenotypes associated with genes [11, 18–22].

We hypothesize that this general framework, and network diffusion in particular, can be extended to aid prioritization of AD genes. Although the underlying biology of AD may be far more diverse than a single function, there are several biological pathways that are aberrantly activated in AD brains, and not surprisingly, most of the genes identified by AD GWAS contribute to these pathways [23]. For example, a primary indicator of AD is the accumulation of amyloid beta plaques in the brain, resulting from mis-processing of *APP* protein [23].

**Fig 1:**
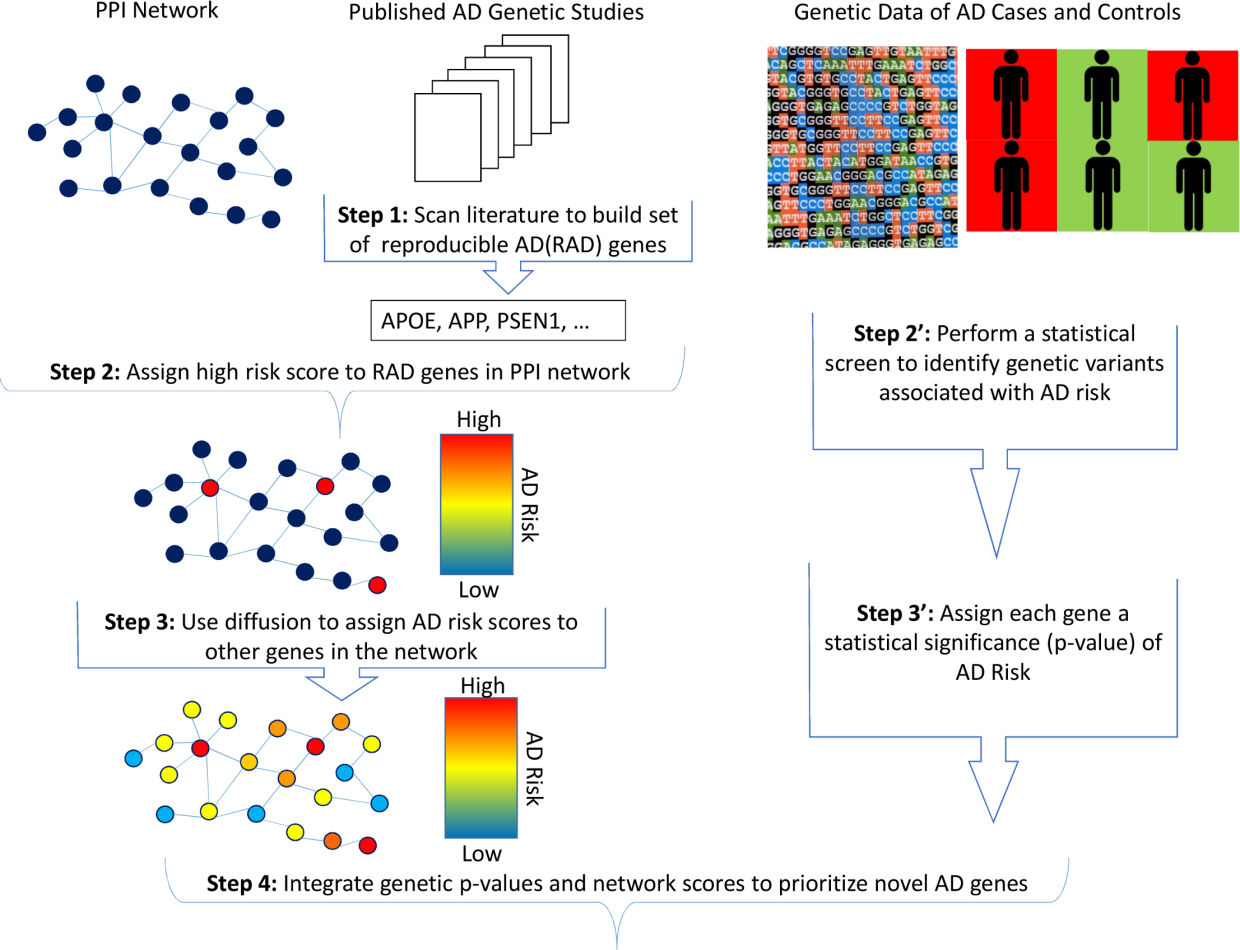
Summary of analysis steps. A set of AD genes that are reproducible (RAD genes) across different genetic studies was assembled through literature curation. The RAD genes were assigned a high initial risk score, and graph theoretical diffusion was employed to derive network diffusion scores for the rest of the genes in the network. Scores obtained from genetic screens and network diffusion were integrated to derive a new prioritization.

We developed a novel re-prioritization approach that can be integrated easily into the current genetic analysis design (**Fig 1**). First, we curated the AD literature to produce a set of approximately 60 robust AD (RAD) genes that includes those that have been associated with AD at the genome-wide significance level or that contain variants shown to affect AD-related processes directly (**Table 1**). We then constructed a network of protein-protein interactions and applied network diffusion to score and rank genes based on their proximity to the RAD genes. Network diffusion allows modeling of indirect interactions, modules and protein complexes that are not modeled if only the direct interactions of proteins are considered. Next, we combined our genetic association results with the network diffusion scores to produce a newly re-prioritized ranking of genes. Finally, we validated our methodology using a novel approach involving bootstrap aggregation on one of the largest assembled genetic datasets of AD. Network-augmented genetic results have measurably improved replication rates in this validation approach. We also show that our main results and key predictions were essentially unchanged after restricting the RAD set to 19 genes which have had been functionally validated as well as replicated in independent datasets.

**Table 1:** RAD genes and the type of study that identified them.

Chr.	Gene	Evidence	Chr.	Gene	Evidence	Chr.	Gene	Evidence
1	***CR1***	GWAS–AD [8, 24]	7	*ZCWPW1*	GWAS–AD [8]	12	*SRRM4*	GWAS–endo [25]
1	***PSEN2***	Linkage [26]	7	***EPHA1***	GWAS–AD [27]	13	*SLCA10A2*	GWAS–AD [8, 28]
2	***BIN1***	GWAS–AD [8]	7	*PLXNA4*	GWAS–AD [29]	14	*FERMT2*	GWAS–endo. [8]
2	*INPP5D*	GWAS–AD [8]	8	*PTK2B*	GWAS–AD [8]	14	***PSEN1***	Linkage [26]
2	*CASP8*	WES [30]	8	***CLU***	GWAS–AD [24]	14	*SLC2A4A*	GWAS–AD [8]
3	*KCNMB2*	GWAS–endo [31]	8	*TP53INP1*	GWAS–AD [32]	14	*PLD4*	GWAS–endo. [33]
3	*OSTN*	GWAS–endo [34]	8	*PDGFRL*	GWAS–endo [35, 36]	15	*TRIP4*	GWAS–AD [37]
4	*UNC5C*	WES [38]	9	*LMX1B*	GWAS–endo [39]	16	***PLCG2***	GWAS–AD [40]
4	*GALNT7*	GWAS–endo [31]	9	*MVB12B*	GWAS–endo	17	***MAPT***	GWAS–AD [41]
5	*MEF2C*	GWAS–AD [8]	10	*ECHDC3*	GWAS–AD [36]	17	*KANSL1*	GWAS–AD [41]
5	*SORCS2*	CGS [9]	10	*SORCS1*	CGS [9]	17	***ABI3***	GWAS–AD [40]
5	*PFDN1*	GWAS–AD [36]	10	*SORCS3*	CGS [9]	17	*ACE*	CGS [42]
6	*HLA-DRB5*	GWAS–AD [8]	11	***CELF1***	GWAS–AD [8]	19	***ABCA7***	GWAS–AD [8]
6	***TREM2***	WES [43]	11	***SPI1***	GWAS–AD [44]	19	*PLD3*	WES [45]
6	*NCR2*	GWAS–endo [34]	11	*MS4A6A*	GWAS–AD [8]	19	***APOE***	Linkage [46]
6	***CD2AP***	GWAS–AD [27]	11	*MS4A4A*	GWAS–AD [8]	19	*CD33*	GWAS–AD [8, 27]
6	*TPBG*	GWAS–AD [36]	11	*MSA6*	GWAS–AD [8, 47]	20	*CASS4*	GWAS–AD [8]
7	*COBL*	GWAS–AD [28]	11	***PICALM***	GWAS–AD [24]	21	***APP***	Targeted Seq. [26]
7	***AKAP9***	WES [48]	11	***SORL1***	CGS [8, 9]	21	*ABCG1*	GWAS–endo. [31]
7	*PILRA*	GWAS–AD [49]	11	*C1QTNF4*	GWAS–endo [25]			

GWAS = genome-wide association study, linkage = family-based linkage study, endo. = AD-related endophenotype, CGS = candidate gene study, WES = whole exome sequencing, target seq. = targeted gene resequencing. Genes that are highlighted in bold text met more stringent criteria and were included in the conservative set of RAD genes.

## Results

### RAD genes are proximal in a PPI network

We assembled a PPI network using interactions pooled from multiple PPI databases (ConsensusPathDB [13], iRefIndex [14], and Human Interactome Y2H [15]) inspired by recent work [21]. Pooling interactions from these three databases resulted in a connected network that includes a large percentage of the genes in our GWAS dataset. We then determined if the RAD genes are proximal within this network. The first proximity measure tested was the average shortest path (ASP) distance [50]. The ASP distance between RAD genes, determined by cross-validation (See Methods), is much smaller than would be expected by random chance (**Table 2**). One problem is that ASP distance between RAD genes and genes with many interactions (the number of interactions a gene has corresponds to its “degree” and high degree genes are considered to be hubs) tends to be small (**Table 3**). In this situation, all hub genes will be falsely predicted to be AD-related. Thus, we incorporated instead the Regularized Laplacian diffusion kernel [51] which penalizes paths going through hubs. The diffusion distance between RAD genes is smaller than would be expected by chance (p = 0.00054) (**Table 2**). Simultaneously, the problematic hub genes in the network have discounted scores as demonstrated by the notable drop in ranking of the 10 genes with the highest number of overall interactions (**Table 3**).

**Table 2:** Proximity between RAD genes in PPI network. Each RAD gene was ranked (in comparison to the other 19,972 genes in the network) based upon its degree (number of interactions in network), its ASP distance to the RAD genes, and total diffusion distance from the RAD genes. The average ranking of the RAD genes was 7,949 using ASP (60th percentile, t-test p = 0.015) and 6,959 for diffusion (65th percentile, t-test p = 0.00054).

Gene	Rank			Gene	Rank			Gene	Rank		
	Degree	ASP	Diffusion		Degree	ASP	Diffusion		Degree	ASP	Diffusion
APP	2	2	1248	MEF2C	3012.5	3072.5	2619	SORCS2	12984	14902.5	1081
CASP8	238.5	76	754	ABI3	3012.5	10739	3228	SORCS3	14153	16106.5	1170
PSEN1	558.5	119.5	441	SORL1	4372.5	9964	2675	ABCG1	14153	7689.5	16627
MAPT	600.5	9	342	TPBG	4516.5	4551.5	5100	TP53INP1	14153	11727	10975
PTK2B	800	175	670	PDGFRL	4862	13192.5	7434	PLXNA4	14153	15296.5	14933
CLU	883	785	1935	LMX1B	5236.5	10441.5	7905	KCNMB2	15703.5	11038.5	12216
PFDN1	930.5	2268	4465	HLA-DRB5	5666.5	4554	7104	SORCS1	15703.5	17153	9425
CD2AP	1043.5	2275.5	585	CD33	5666.5	2281.5	1682	MS4A6A	15703.5	19883.5	19955
PSEN2	1188	454	642	PLD3	5891.5	4554.5	4320	ABCA7	15703.5	7689.5	17609
AKAP9	1230	4547.5	2996	CELF1	5891.5	789	3793	SRRM4	18290	18462.5	18934.5
PLCG2	1255	281	868	PILRA	6640.5	13274.5	8762	CASS4	18290	14847.5	16647.5
APOE	1517	283	626	CR1	7296.5	15652	12460	ECHDC3	18290	19700.5	19390
INPP5D	1582	455	795	GALNT7	7296.5	7688	8782	PLD4	18290	7689.5	17433
BIN1	1691	457	977	MVB12B	7995.5	7688.5	4498	TREM2	18290	19587	1566
TRIP4	2509	4548.5	5679	ACE	8878	4555	9212	SLC10A2	18290	7689.5	17128
PICALM	2640	3070.5	1207	EPHA1	9380.5	7689	8437	ZNF804B	18290	18465	18406
KANSL1	2780	3069.5	3734	COBL	9928.5	13930	9416	NCR2	18290	19587	1566
FERMT2	2857.5	1496.5	3313	UNC5C	12984	14796.5	15064				

**Table 3:** Proximity of non-RAD hub genes to RAD genes.

	Rank		
Gene	Degree	ASP	Diffusion
UBC	1	1	1433
SUMO2	2	20.5	1570
CUL3	3	51	2515
SUMO1	4	20.5	1502
EGFR	5.5	3	937
TP53	5.5	7	983
GRB2	7	2	905
SUMO3	8	181	2433
HSP90AA1	9	10	978
MDM2	10	51	1096

### Filtering by network diffusion score improves replication rate

**Fig 2:**
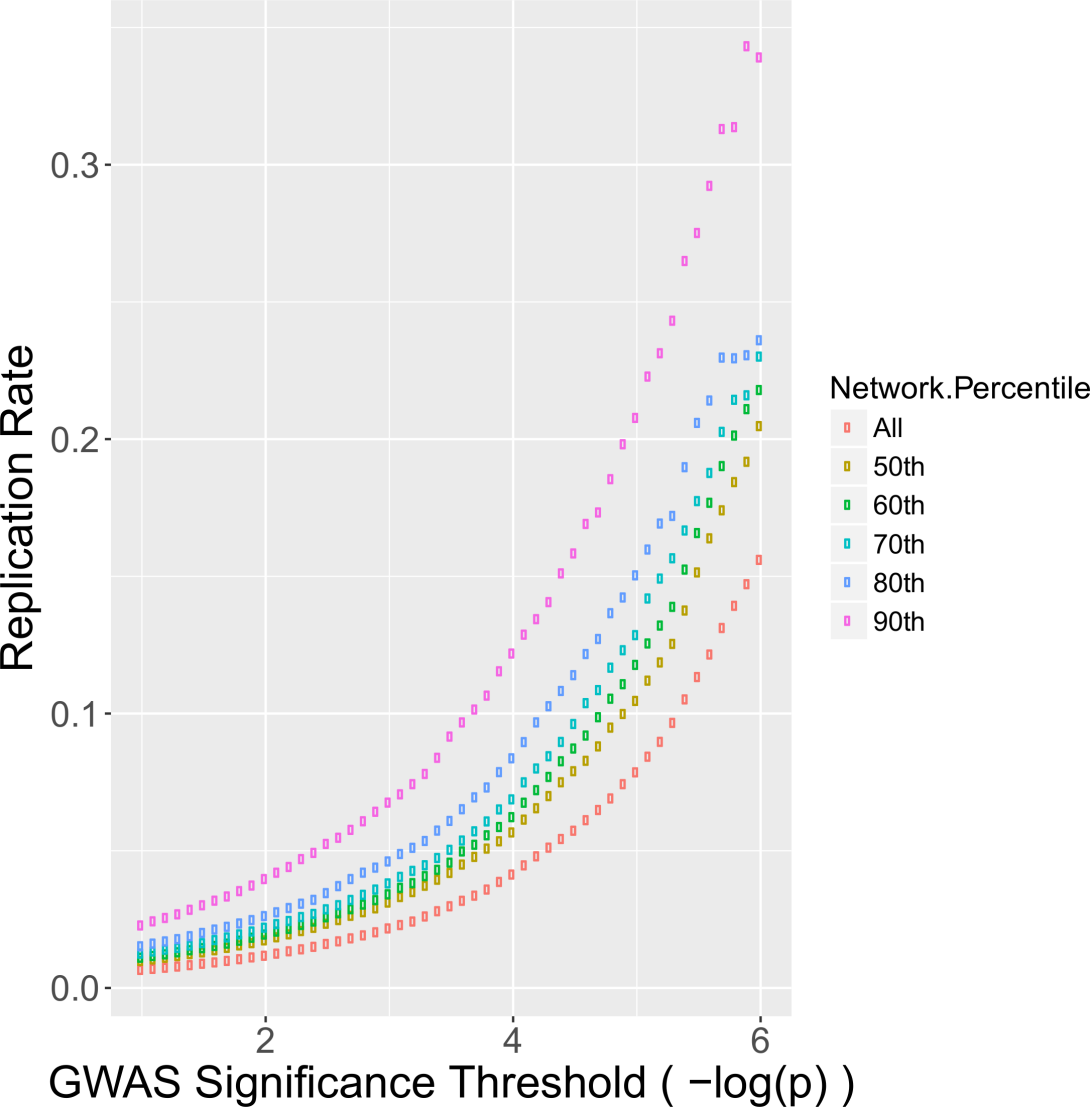
Filtering on network score improves replication rate. The replication rate was computed for all genes surpassing the significance threshold for each GWAS. This procedure was repeated in each bootstrapped dataset and the average replication rate was determined (purple). This process was repeated using increasingly strict filters on the network diffusion scores. The baseline replication rate without utilizing network scores (naïve method) is represented by the purple points. The strictest network filter (red) has a consistently higher replication rate than the naïve method.

We next tested if genes with high diffusion scores replicate more frequently in order to demonstrate that diffusion scores are informative when used in conjunction with genetic data. Bootstrap aggregation [52] was applied to our genetic dataset to produce a large number of pairs of discovery and replication datasets (See Methods). In each discovery + replication pair, we conducted a standard genetic workflow, beginning with a screen in the discovery dataset followed by validating top findings in the replication dataset. For each pair, a replication rate was calculated by determining the percentage of genes that surpass a given significance threshold also replicated. To test if network diffusion scores improved replication, we altered the standard discover + replication approach. We ranked genes by their network diffusion score and then iteratively dropped genes that had ranking diffusion scores below a given stringency threshold. At first we retained only genes in the 50th percentile of network scores, then gradually increased the threshold to only include genes in the 60th, 70th, 80th, and 90th percentiles. For each threshold, we computed the replication rate and compared to the baseline. As shown in **Fig 2**, filtering based upon network score percentile noticeably increased replication rate. Genes with a–log(p-value) of > 6 replicated at a rate of approximately 16% in simulations (farthest right purple point), while additional strict network filtering improved the replication rate to nearly 34% (farthest right red point).

### Combined Z-scores predict novel AD genes

**Fig 3:**
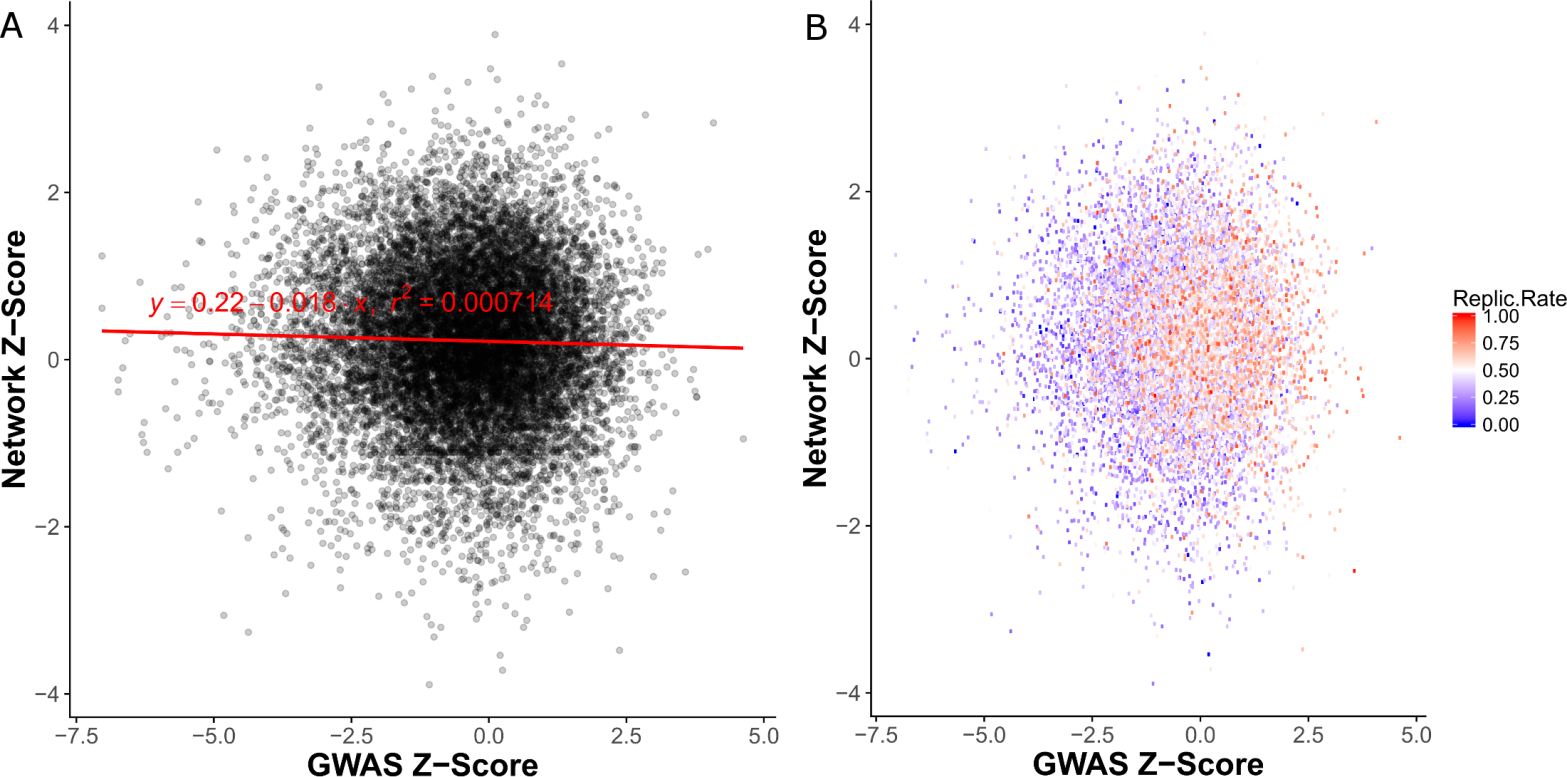
Comparison of GWAS and network Z-scores. **A.** Transformed Z-scores are uncorrelated. **B.** Genes with high network scores had higher replication rates compared to those with low network scores, as further visualized and confirmed statistically as shown in **Fig 4**. Reprate = replication rate.

Since filtering on network diffusion score improved replication rate, we next sought to integrate the network diffusion scores and genetic results into a single score. First, we converted the p-value of each gene from genetic analysis into a Z-score (“GWAS Z-Scores”) and then converted the network diffusion percentile of each gene into a Z-score (“Network Z-scores”). Linear regression analysis showed that the Network and GWAS Z-scores are independent (**Fig 3A**). Next, we assigned each gene a replication rate based upon how frequently the gene replicated in our bootstrapped validation datasets (See Methods). We observed that replication rates were higher for genes with higher network Z-scores compared to genes with lower network Z-scores (**Fig 3B**).

**Fig 4:**
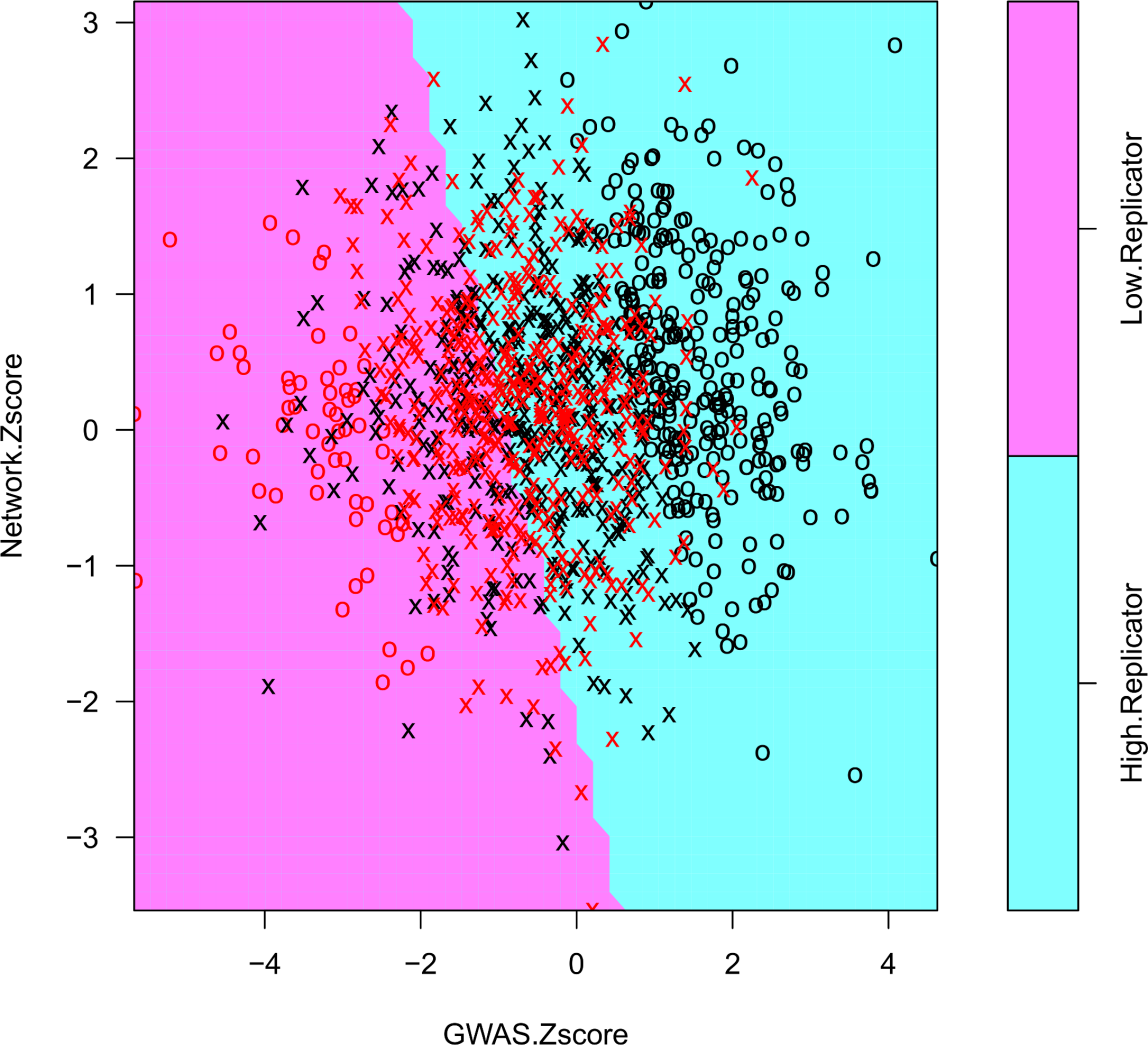
Support vector machine training to predict GWAS and network Z-score weights. Selection of genes with a high replication rate (> 0.7, blue points) and low replication rate (<0.1, red points) yielded a balanced number of genes in each replication class (high/low). A linear SVM model was trained to predict replication class using the GWAS and network Z-scores of each gene. Genes represented as X's were used as support vectors for the training of the SVM, whereas genes represented as O's were not. Both network and GWAS Z-scores contributed to the decision boundary, as demonstrated by the significance of their predicted coefficients using logistic regression (GWAS: p <2.0×10^−16^, Network: p = 0.0016).

To combine the Network and GWAS Z-scores, we developed an approach that uses a linear support vector machine (SVM) [53] to determine how heavily each type of score should be weighted in order to maximize replication rate (See Methods). These weights were then used in conjunction with the meta-analysis method for combining summary results implemented in METAL [54]. The weights predicted by the SVM (**Fig 4**) were 0.703 (GWAS) and 0.297 (Network). As further confirmation, we conducted binomial (logit family) logistic regression using network and GWAS Z-scores as predictors and the replication class (high/low) as the outcome. Both network and GWAS score were significant, (GWAS: coefficient = -0.659, p <2.0×10^−16^) (Network: coefficient = -0.229, p = 0.0016). The coefficients derived from logistic regression are very similar to the SVM-derived weights (GWAS weight = 0.742, Network weight = 0.258).

Next, we applied our combined approach genome-wide, excluding the RAD genes and genes containing significantly associated variants (p <1.0x10^-7^) to focus on novel candidates. Among the genes with largest combined Z-scores (**Table 4, S1 Table**), several have important roles in inflammation. *CR2* (p = 5.95×10^−7^) is a receptor protein involved in immune response (genecards.com [55]). *SHARPIN* (p = 1.43×10^−5^) is a component of the LUBAC complex that plays a regulatory role in inflammation [55]. *PTPN2* (p = 3.21×10^−5^) is a phosphatase that also serves an important role in regulation of inflammation and glucose homeostasis [55]. The Bonferroni-corrected significance threshold when considering only genes in the 75^th^ percentile of network scores is p = 1.46 x 10^−5^, although this is likely to be overly strict since proximally located genes are not inherited independently.

**Table 4:** Top predicted AD genes using combination approach.

	Z-Score		
Gene	GWAS	Network	Combined
CR2	4.084	2.832	4.857
SHARPIN	3.983	1.320	4.185
PTPN2	3.805	1.259	3.997
C4B	2.846	2.928	3.750
TUBB2B	3.166	1.314	3.428
EPS8	3.156	1.156	3.358
PSMC3	3.145	1.036	3.302
STRAP	3.051	1.157	3.262
HSPA2	2.977	1.325	3.258
STUB1	2.895	1.407	3.213

We performed pathway analysis using Gene Set Enrichment Analysis (GSEA) [56] to determine if AD-related pathways are more enriched when genes are ranked by their combined Z-scores versus GWAS-only Z-scores (See Methods). Notably, ranking genes based upon combined Z-scores resulted in several significantly enriched AD-related pathways including immune response, FOX03 targeting (indicates enrichment for aging), and hippocampal development (**Table 5**). By comparison, ranking genes based only upon their GWAS Z-scores resulted in virtually no significant pathways entirely (**Table 6**).

**Table 5:** GSEA results after ranking genes by combined Z-scores.

PATHWAY NAME	SIZE	ES	NES	FWER p-val
KEGG_ANTIGEN_PROCESSING_AND_PRESENTATION	64	0.487	2.231	0.042
DELPUECH_FOXO3_TARGETS_DN	37	0.527	2.181	0.07
BIOCARTA_PGC1A_PATHWAY	20	0.613	2.180	0.071
KEGG_SYSTEMIC_LUPUS_ERYTHEMATOSUS	85	0.436	2.171	0.073
MURAKAMI_UV_RESPONSE_6HR_DN	20	0.592	2.124	0.117
GOLUB_ALL_VS_AML_DN	18	0.629	2.118	0.127
REACTOME_RNA_POL_I_PROMOTER_OPENING	28	0.552	2.100	0.149
MODY_HIPPOCAMPUS_PRENATAL	36	0.519	2.098	0.153
FARMER_BREAST_CANCER_CLUSTER_5	17	0.632	2.090	0.161
ZUCCHI_METASTASIS_DN	35	0.516	2.067	0.197
NAKAYAMA_SOFT_TISSUE_TUMORS_PCA1_UP	61	0.456	2.058	0.205
INGA_TP53_TARGETS	15	0.635	2.049	0.222

**Table 6:** GSEA results after ranking genes by GWAS only Z-scores.

PATHWAY NAME	SIZE	ES	NES	FWER p-val
NAKAYAMA_SOFT_TISSUE_TUMORS_PCA1_UP	61	0.440	2.108	0.134
FARMER_BREAST_CANCER_CLUSTER_5	17	0.610	2.016	0.261
KEGG_ANTIGEN_PROCESSING_AND_PRESENTATION	64	0.397	1.950	0.418
GOLUB_ALL_VS_AML_DN	18	0.560	1.888	0.591
CHIARETTI_T_ALL_REFRACTORY_TO_THERAPY	23	0.499	1.879	0.608
SHIN_B_CELL_LYMPHOMA_CLUSTER_5	15	0.541	1.772	0.864
ZUCCHI_METASTASIS_DN	35	0.423	1.769	0.873
KIM_HYPOXIA	22	0.456	1.704	0.964
DELPUECH_FOXO3_TARGETS_DN	37	0.400	1.672	0.985
NIELSEN_LIPOSARCOMA_UP	15	0.514	1.650	0.993
ROVERSI_GLIOMA_COPY_NUMBER_UP	56	0.351	1.643	0.996

## Discussion

GWAS of AD and AD-related endophenotypes have discovered and replicated associations with more than 60 genes (**Table 1**), many of which have roles in AD-related pathways (amyloid β aggregation, inflammation, cholesterol transport, immune response, etc.). To identify additional AD-related genes, we hypothesized that genes having suggestive evidence for association from a genome-wide screen and protein-level interactions (both direct and indirect) are more likely to replicate. This idea has been referred to as functional linkage [57]. To test this hypothesis, we developed a novel approach for improving the prioritization of candidate disease genes that incorporates a network diffusion of scores from known disease genes using a protein network and integration with GWAS risk scores. We tested this approach on a large AD GWAS dataset and validated the performance of the methodology using bootstrap aggregation. Several novel AD genes were predicted including *CR2*, *SHARPIN*, *and PTPN2*.

Part of the motivation for our approach was to identify genes that are more obviously biologically relevant to AD. This is exemplified by *SHARPIN*, whose principal known function is to form the LUBAC complex and prevent inflammation, a major process through which amyloid aggregation and AD are thought to develop [23]. Similarly, *CR2*, a homolog of *CR1* which is a well-established AD gene [8], is involved in immune response. Many immune response genes are differentially expressed between healthy and AD brains, and investigations into the connection between expression in cell types and the presence of AD has led to growing interest in the role microglial cells (a first responder in the immune response pathway) [58]. Finally, *PTPN2* is involved in multiple AD-related pathways; it has roles in negatively regulating inflammation and de-phosphorylation of key glucose metabolism kinases including *INSR* and *EGFR* [59]. The AD-related roles of each of our novel AD gene predictions, in combination with their strong network and genetic scores, make them highly promising candidates.

One biological form of functional linkage that does not require direct physical interaction is membership in the same signaling pathway or protein complex. For example, our study identified interaction between *FOXO* and *INSR* that is consistent with evidence of a multi-link signaling pathway comprised of direct physical interactions in the insulin-signaling pathway [60]. By comparison, neighborhood enrichment approaches (i.e., testing a gene’s direct interactions) cannot detect indirect interactions. Furthermore, neighborhood enrichment approaches are unreasonable for AD because some RAD genes are network hubs (e.g., *APP* has more than 2000 interactions) which would result in an unreasonably high number of genes having AD-enriched neighborhoods.

Some distance metrics capture indirect interactions by calculating the proximity between a pair of genes based upon short paths between them in the network. However, after testing a simple distance metric known as average shortest path (ASP), we observed that hub genes were still the top-ranked predicted genes. Since hub genes have many interactions, they tend to have short overall paths to any genes in a network, although their functions are highly generic and unlikely tied to a particular disease. Ubiquitin C (*UBC*), for example, has nearly 9,000 interactions; however, this is simply because protein degradation is essential for regulating the vast majority of proteins. Therefore, a more nuanced network propagation approach can aid in making disease specific inferences.

Network diffusion is a widely used class of spectral graph clustering methods that have been applied to many computational disciplines [51]. We used this approach to propagate evidence in the form of AD scores throughout the network. A protein in the network that has a short “diffusion distance” to one or more well-established AD genes will receive a high network risk score. Notably, we observed that network diffusion down-weights hubs while simultaneously outperforming ASP distance when applying leave-one-out cross-validation to the RAD genes. Many diffusion kernels have been proposed in graph theory, however the Regularized Laplacian [51] approach used in this study has the highly desirable properties of requiring very little parameterization (in fact, only a single parameter is required to be set) and also more computationally efficient than other diffusion kernels. Network diffusion methods have been applied in other genetics research contexts such as labeling somatic network mutations in cancer [61], characterizing gene sets [62], and predicting risk genes for amyotrophic lateral sclerosis [21].

We also observed that genes with high diffusion scores tended to replicate more frequently in our 125 pairs of bootstrapped discovery and replication datasets. However, network Z-scores and GWAS Z-scores in the full dataset were not strongly correlated. Taken together, these observations indicate the importance of considering jointly protein interaction data and genetic results even though they are independent because the integration of both types of information will likely yield noticeable improvement in replicability of findings. Since our bootstrapping procedure required splitting the original dataset, the simulations were conducted using datasets that contained only one-half of the total sample. This suggests that our network scores aided in determining which genetic associations were real in datasets with reduced power. We note that our bootstrapping approach was performed on the same data from which we derived the GWAS Z-scores used to train the SVM. Therefore, the selection of combination weights may have been biased in favor of GWAS Z-scores. Furthermore, it is unclear whether the weight combination used in this study (0.297/0.703) would be appropriate for combining genetic and network data for other disorders or traits.

The GWAS approach has a very limited capability to identify the entire set of genes which contribute to the risk of a complex disease like AD, even in datasets containing up to 100,000 individuals, because some genes do not contain variants that are sufficiently frequent and/or exert a large enough effect to yield a statistically significant association. To overcome this limitation, we developed a novel SVM approach to integrate the genetic and network scores by propagating GWAS Z-scores in a PPI network. In the AD example presented here, we initialized the RAD genes to have an identical high score in the network, thereby allowing re-prioritization of genes in any AD dataset regardless of the internal Z-scores of the RAD genes.

We acknowledge that our initial choice to treat each RAD gene equally may be controversial. Arguably, we could have seeded our analyses with GWAS Z-scores for each RAD gene from the original studies. However, our approach permits unbiased exploration of interactions of all plausible AD genes and does not require adjustment to these Z-scores for sample size or allele frequencies. Moreover, results derived from weighted RAD genes would be dominated by interactions with *APOE* for which the significance level exceeded a–log(p-value) of more than 100 in several datasets (compared to < 10 for most other RAD genes in the total group of datasets). Also, several key AD-related genes (e.g., *APP*, *PSEN1* and *PSEN2*) which show little evidence for association with individual SNP or gene-based tests for AD would be undervalued in analyses using weighted Z-scores. In order to make our software maximally flexible and support weights derived from confidence in the seed genes, we implemented an option for users to specify unequal weights on the seed genes at their own discretion.

A potential concern about our results is the strategy for selecting RAD genes because many significant GWAS findings include variants located in intergenic regions. The most parsimonious explanation is that the variant responsible for the association peak influences the nearest gene, but there is abundant evidence suggesting this assumption is often incorrect. To address this issue, we repeated our analyses using a more restricted set of RAD genes that included only those supported by genome-wide significant evidence of association with AD risk and replication in independent datasets or by other genetic evidence plus experiments linking them to AD-related pathophysiology. Our leave-one-out cross validation approach demonstrated that the genes in the restricted RAD set had closer network proximity to each other than would be expected by chance (p = 5.93x10^-5^, **S2 Table**). The statistical support for the novel genes *CR2* (p = 4.09x10^-7^), *SHARPIN* (p = 1.10x10^-5^), and *PTPN2* (p = 2.41x10^-5^) remained the same (**S3 Table**). Finally, combined Z-scores that were derived using diffusion from the more conservative RAD gene set yielded similar AD-related pathways such as Fx03 targets (FWER p = 0.064), antigen processing (FWER p = 0.02), and hippocampal development (FWER p = 0.065) (**S4 Table**). These results confirm that the genes with a clear functional role in AD produce network diffusion-based predictions that are consistent with the results presented here. Curiously, the inclusion or exclusion of the portion of RAD genes that have an ambiguous or non- validated functional role in AD did not affect our results.

We also acknowledge that several of the novel putative AD genes may have been erroneously prioritized because they are in the same locus with RAD genes. This concern is unlikely noting that there are several instances where a genetic association peak includes multiple genes that may have a possible functional role in AD (e.g., the *MS4A* gene cluster [8]). Although one of our novel AD genes, *CR2*, is located close to *CR1*, which is an unambiguous RAD gene given its robust replication in GWAS and effect on deposition of neuritic amyloid plaque [63], *CR2* is also an intriguing AD candidate gene because it has been shown to regulate hippocampal neurogenesis [63]. Thus, our findings suggest that our approach will aid in predicting truly multiple AD-related genes at a locus, however additional biological evidence may be required in some instances to make this distinction.

Previous AD studies have implicated inflammation and immune response genes, but we did not observe enrichment for these pathways when incorporating only GWAS scores in the analysis. However, these and other recognized AD-related pathways emerged after applying our network re-prioritization method (Table 6) suggesting that incorporation of network data can help minimize discrepancies in predictions across different genetic datasets. On the other hand, other well-established AD-related pathways, including cholesterol metabolism and endocytosis, were not detected by our approach. Further inspection of the results revealed, for example, that enrichment for the cholesterol homeostasis pathway is not significant when applying GSEA to the genetic data only (FWER p = 1). This pathway as defined in the Molecular Signatures Database (MSigDB) is very broad and contains many genes that are weakly associated with AD which consequently diminish the enrichment of the set. The evidence for this pathway is greater in the analysis using only network scores (FWER p = 0.18), which indicates our method still improves the detection of cholesterol homeostasis. Even pathways such as HDL-mediated lipid transport that were enriched in analyses considering only genetic data (largely due to the strong signal from APOE) were not ranked highly by our network diffusion algorithm because RAD genes such as APOE are ignored to minimize bias.

Although merging of multiple databases to obtain a very highly connected network is a requirement for the diffusion algorithm to work properly, our approach offers several advantages in comparison to other network-based approaches including biological transparency, ease of integration with a variety of GWAS methods, and the ability to balance data-driven statistics and biological prior probabilities. The extensive simulations we conducted provide a general basis for further establishing the practicality of genetic and network-based integration. Our network methodology was developed with the goal of accommodating known complications of genetic analysis.

The software developed for this study is open source, accessible to most users (incorporated in an R package), and applicable to any set of variant- or gene-level disease association results. Importantly, it requires only a set of GWAS results and a list of previously known disease genes and, therefore, does not necessitate changes to previously established genetic analysis pipelines. Although we used an SVM procedure to determine the weights for the score combination, a user can specify any weights or simply use our defaults that are based on the 0.297/0.703 ratio determined by SVM. Our package is accessible through GitHub (https://github.com/lancour/ignition).

## Methods

### Assembling an AD gene list

A set of genes ascribed to AD with a high degree of certainty was assembled through curation of published findings ascertained through PubMed searches that emerged from studies using a variety of approaches including GWAS of AD risk and AD-related endophenotypes, family-based linkage analysis, positional cloning, whole exome sequencing (WES), and candidate gene testing (CGS) (Table 1). Criteria for inclusion in this set included (1) genome-wide significance for GWAS and WES studies (p < 5x10^-8^) and LOD score > 3 for linkage studies and (2) replication of association signals in independent datasets; or (3) biological evidence that demonstrate functional relevance to AD of associated variants or the encoded protein.

### Harmonizing protein-protein interaction databases

A set of interacting gene-gene pairs (in HGNC symbol format) is required as input for this software. To compile this set, three databases (RefIndex v14 [14], ConsensusPathDB v31 [13], and Human Interactome Y2H DB vHI-II-14 [15]) were selected based on their demonstrated utility in recent work [21]. iREFINDEX and ConsensusPathDB interactions were filtered to remove self and complex (more than two proteins) interactions. The ConsensusPathDB interactions are given in uniProt ID format, which were converted to HGNC symbols using the official website (http://www.genenames.org). iREFINDEX provides a HGNC symbol for each interactor of an interaction when possible, and so only interactions which had a HGNC for both interactors were kept. The Human Interactome DB already provides a set of binary gene-gene interactions in HGNC format, so no processing was required. The union of the processed sets from each database was used as the final interaction set. The unified set contains 19,972 unique gene symbols and 236,642 interactions. These databases are curated collections of experimentally determined interactions (typically binding or affinity) reported in the literature, such as from co-immunoprecipitation, as well as predicted interactions in a small number of databases.

### Assigning network scores to genes through diffusion

Network diffusion is a very well-studied spectral approach to graph clustering and annotation [17, 51, 64, 65]. It attempts to mimic node-to-node distance in the graph that in turn aims to capture functional relevance. The first step of the diffusion method is to model the protein interactions as a network. A network is comprised of a set of nodes, *V*, and a set of edges between nodes, *E*. For this work, nodes represent genes, and edges represent an interaction present in the unified set. Although we use unweighted edges in this work, our network methods and software are able to receive weighted input as well, such as protein interactions with confidence measures taken from STRING [66]. The construction of diffusion kernels using weighted edges has been well studied and is equally valid [51]. *n* is the number of nodes in the network, which is 19,972 (yielding 236,642 edges). All network methods were implemented in R. The regularized Laplacian kernel [51] is constructed by:

$$K={\left(I+\alpha L\right)}^{-1}$$ (1)

where *K* is the resulting kernel, *I* is the identity matrix, *L* is the graph Laplacian, and alpha is a constant (see **S1 Text** and [51] for additional details). For this study, an alpha value of 0.1 was used, consistent with other work in this field [17]. Next, a network diffusion score was computed for each gene. To do this, the diffusion score vector, *y*, was initialized to be a length *n* vector that contains 1’s in the indices of the RAD genes, and 0’s otherwise. Risk scores for all genes in the graph were then derived by multiplication of *K* by the diffusion score vector *y*: ỹ = *Ky*.

### Validation of diffusion approach using leave-one-out cross validation

To test if RAD genes had closer than random diffusion proximity to other RAD genes in a network, leave-one-out cross validation [67] was applied to the RAD gene set. First, a single RAD gene from the RAD set was set to 0 in the initial diffusion score vector, *y*. Then, diffusion scores were computed based upon this new initialization of *y*. The diffusion scores were sorted and the sorted rank of the removed RAD gene’s diffusion score was determined in comparison to all other non-RAD genes. This process was repeated for each gene in the RAD set, resulting in a list of ranks. If diffusion proximity is informative and potentially predictive, the average rank of the RAD genes should be significantly lower than the average rank of all genes, (n+1) / 2, which was verified using a one-tailed t-test.

### ADGC GWAS dataset

The Alzheimer’s Disease Genetics Consortium (ADGC) is an NIA-funded project whose goal is to identify genes associated with an increased risk of developing late-onset Alzheimer disease (LOAD) by assembling and analyzing genetic and phenotypic data from large cohorts containing rigorously evaluated AD cases and cognitively normal controls of various ethnic ancestries. Details of ascertainment, collection, quality control (QC), and analysis of genotype and phenotype data in the individual datasets of the ADGC are provided elsewhere [8, 68]. Here we examined genotype data that were generated using high-density SNP microarrays from 32 prospective, case-control, and family-based studies of LOAD comprising 16,175 case and 17,176 controls of European ancestry. After QC steps to filter low-quality SNPs and individuals with low genotype call rates, principal components (PCs) of ancestry were computed within each dataset using EIGENSTRAT [69] and a set of 21,109 SNPs common to all genotyping platforms and datasets in order to account for population substructure in genetic association analysis. Samples with outlier PC values >six standard deviations from the mean were excluded from subsequent analyses. Genotypes for a much larger set of SNPs were imputed using the Haplotype Reference Consortium panel release 1.1 [70, 71], which includes 64,976 haplotypes derived from 39,235,157 SNPs, and the Michigan Imputation Server (https://imputationserver.sph.umich.edu/) running MiniMac3 [72, 73].

### Genome-wide association analysis

Association of AD with the imputed dosage of the minor allele for each SNP (a quantitative estimate between 0 and 2) genome-wide was conducted using logistic regression models implemented in PLINK [74] that included covariates for age-at-onset/age-at-exam, sex, the first three PCs, and an indicator variable for each dataset. Joint analysis was chosen in favor of meta-analysis to avoid problems that could be introduced if bootstrap aggregation under-sampled small cohorts, resulting in unreliable association estimates for those cohorts. To account for relatedness in family datasets, subsets of maximally-unrelated affected and unaffected individuals were sampled from each pedigree. Each variant was annotated to a gene region according to RefSeq release 69 [75] using the program ANNOVAR [76]. Then, each gene was assigned the minimum p-value of all variants annotated to it, after applying the following formula:

$$P_{g}^{{Gene}^{\prime }}=1-{\left(1-P_{g}^{BestSNP}\right)}^{\frac{N+1}{2}}$$ (2)

where N is the number of variants analyzed that were annotated to the gene. Previously, this correction [77] has been shown to perform comparably to more complex adjustments based upon gene length, recombination hotspots, and similar gene features [78].

### Validation of genetic re-prioritization through bootstrap aggregation

Since the availability of large AD genetic datasets is limited, bootstrap aggregation [52] was used to generate a high number of datasets for method validation. First, the full ADGC dataset was equally separated into discovery and replication halves. Then, 25 iterations of bootstrap aggregation were applied to the discovery half and then the replication half. The resultant 25 discovery and 25 replication datasets were then matched (D1 and R1, D2 and R2….D25 and R25). To further ensure robustness, the splitting procedure was repeated a total of 5 times, with 25 iterations of bootstrap aggregation applied each time, resulting in 125 total pairings (D1 and R1, D2 and R2. …D125 and R125). Each pairing represents a discovery dataset as well as an independent replication dataset.

For each pairing, the previously described genetic analysis was conducted on the discovery half. Then all genes that passed a designated significance threshold (the number of passing genes is denoted as *r*) were selected to be tested again in the replication half using a significance threshold of (0.05 / *r*). The replication rate was computed by determining the percentage of passing genes in the discovery half that also passed in the replication half. A replication rate was estimated for each pairing, and the mean replication rate was then determined. Next, the replication rate was re-determined for each pairing, with the added criterion that selected genes must also have a top percentile network diffusion score (top 10th, 20th, 30th, 40th, and 50th were tested). The average replication rate for each filtering threshold was compared to the average replication rate without filtering.

### Integrating GWAS and network diffusion scores

The p-values from genetic analysis of the ADGC dataset were converted to Z-scores using the qnorm function in R. Then, the network diffusion scores were converted into percentiles. The percentiles are transformed into Z-scores using the qnorm function, with the additional specification of lower.tail = F. The weighting scheme from METAL was applied to combine the GWAS and network Z-scores:

$$Z_{combined}=\frac{w_{1}*Z_{gwas}+w_{2}*Z_{network}}{\sqrt{w_{1}^{2}+w_{2}^{2}}}$$ (3)

Although any weight selection can be used, the weights were “learned” using an SVM [53] due to the observation that the GWAS and network scores did not contribute equally to predicting replication rate. First, a replication rate was determined for each gene. If a gene had a p-value of <0.05 in *d* discovery datasets and a replication p-value of <0.05 in *r* of the paired replication datasets, it was assigned a replication rate of *r/d*. To reduce model overfitting, create sufficient separation between the classes, and achieve a balance of high and low replicating genes, only high replication genes (≥0.7, n = 676) and low replication genes (<0.1, n = 475) representing approximately 8.4% of the total genes with both a network and GWAS scores were extracted. By comparison, using a threshold of 0.8 or 0.9 would result in an imbalanced training set with very few high replication genes because highly replicating genes are uncommon. A linear SVM [53] was trained using the network Z-scores and the genetic association Z-scores as features, and “high” and “low” as the classes. The resulting slope of decision boundary was then used to determine appropriate weights (w_1_ = 0.703, w_2_ = 0.297).

### Pathway analysis using the re-prioritized ordering of genes

Pathway enrichment was performed using the Gene Set Enrichment Analysis (GSEA) software [56]. GSEA’s pre-ranked analysis tool requires that the user provide a numeric measure for ordering genes. To establish a baseline, enrichment was done using our internal GWAS Z-scores to order genes. Then, enrichment was done using the alternative ordering genes based upon their combined Z-scores (see above for combination method). The gene sets tested for enrichment were the GSEA C2 pathways in MSigDb, which are the “curated gene sets” compiled from multiple sources including KEGG [60], Reactome [79], and domain experts. The significance threshold was set at FDR < 0.25, as suggested previously for this hypothesis generating approach [56].

### Ethics statement

The use of de-identified human subject information for this study was approved by the Boston University Institutional Review Board.

## Supporting information

S1 Table Top predicted AD genes using combination approach. (XLSX)

S2 Table Leave-one-out cross validation rankings using the conservative RAD set. (XLSX)

S3 Table Top combined predictions using the conservative RAD gene list. (XLSX)

S4 Table GSEA results based on conservative RAD gene set after ranking genes by combined Z-scores. (XLSX)

S1 Text Regularized Laplacian construction. (DOCX)
