# Supplementary material for: One for all and all for One: Improving replication of genetic studies through network diffusion
Source: PLoS Genet. 2018 Apr 23;14(4):e1007306. doi: 10.1371/journal.pgen.1007306 (PMC5933817; doi:10.1371/journal.pgen.1007306)

Regularized Laplacian Construction

The adjacency matrix, *A,* is as an *n* x *n* matrix (each row and column represents a gene) with entries defined by:


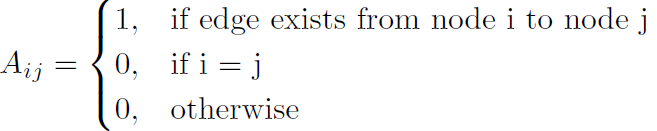


The degree matrix, *D*, is a diagonal matrix containing the total number of edges each node has:


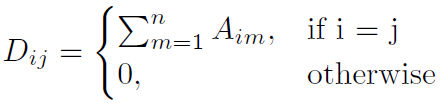


The graph Laplacian, *L,* is then:

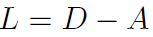


Now the Regularized Laplacian kernel is constructed, which is common kernel for modeling diffusion in other contexts:


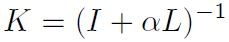


Alpha is a scalar that acts as a decay factor on the diffusion. An alpha value of 0.1 is used, consistent with other work in this field. Now a network diffusion score is assigned to each gene. To do this, the diffusion score vector, *y*, is initialized to be a length *n* vector that contains 1’s in the indices of the RAD Genes, and 0’s otherwise. We then compute risk scores for all genes in our graph through matrix multiplication:


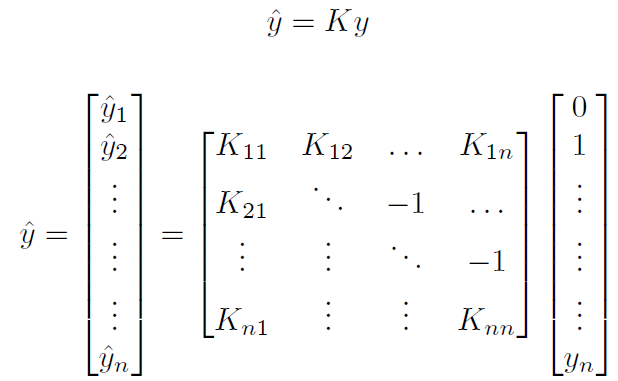

Supplement: S1 Text — (DOCX) [file pgen.1007306.s005.docx]
